# Chronic Sulfasalazine Treatment in Mice Induces System x_c_^−^ - Independent Adverse Effects

**DOI:** 10.3389/fphar.2021.625699

**Published:** 2021-05-18

**Authors:** Lise Verbruggen, Lindsay Sprimont, Eduard Bentea, Pauline Janssen, Azzedine Gharib, Lauren Deneyer, Laura De Pauw, Olaya Lara, Hideyo Sato, Charles Nicaise, Ann Massie

**Affiliations:** ^1^ Laboratory of Neuro-Aging & Viro-Immunotherapy, Vrije Universiteit Brussel, Brussels, Belgium; ^2^ Laboratory Neurodegeneration and Regeneration, URPHyM-NARILIS, Université de Namur, Namur, Belgium; ^3^ Department of Medical Technology, Niigata University, Niigata, Japan

**Keywords:** system x_c_^−^, sulfasalazine, adverse (side) effects, spinal cord, behavior, cystine, glutamate

## Abstract

Despite ample evidence for the therapeutic potential of inhibition of the cystine/glutamate antiporter system x_c_^−^ in neurological disorders and in cancer, none of the proposed inhibitors is selective. In this context, a lot of research has been performed using the EMA- and FDA-approved drug sulfasalazine (SAS). Even though this molecule is already on the market for decades as an anti-inflammatory drug, serious side effects due to its use have been reported. Whereas for the treatment of the main indications, SAS needs to be cleaved in the intestine into the anti-inflammatory compound mesalazine, it needs to reach the systemic circulation in its intact form to allow inhibition of system x_c_^−^. The higher plasma levels of intact SAS (or its metabolites) might induce adverse effects, independent of its action on system x_c_^−^. Some of these effects have however been attributed to system x_c_^−^ inhibition, calling into question the safety of targeting system x_c_^−^. In this study we chronically treated system x_c_^−^ - deficient mice and their wildtype littermates with two different doses of SAS (160 mg/kg twice daily or 320 mg/kg once daily, i.p.) and studied some of the adverse effects that were previously reported. SAS had a negative impact on the survival rate, the body weight, the thermoregulation and/or stress reaction of mice of both genotypes, and thus independent of its inhibitory action on system x_c_^−^. While SAS decreased the total distance travelled in the open-field test the first time the mice encountered the test, it did not influence this parameter on the long-term and it did not induce other behavioral changes such as anxiety- or depressive-like behavior. Finally, no major histological abnormalities were observed in the spinal cord. To conclude, we were unable to identify any undesirable system x_c_^−^-dependent effect of chronic administration of SAS.

## Introduction

System x_c_^−^ is a cystine/glutamate antiporter with xCT (Slc7a11) as specific subunit and is located mainly in the central nervous system and peripheral organs related to the immune system. xCT expression is enhanced in conditions of increased oxidative stress and/or inflammation, and inhibition of system x_c_^−^ has been proposed as a treatment strategy for several neurological disorders as well as for diverse cancer types (Lewerenz et al., 2013; Massie et al., 2015; Koppula et al., 2018; Liu et al., 2020). However, despite many attempts, till now none of the available inhibitors are selectively targeting system x_c_^−^. Preclinical research is as such hampered by the lack of specific tools to interfere with the function of system x_c_^−^ *in vivo* as it is difficult to distinguish the off-target effects of the non-specific inhibitors from the effects that are mediated by inhibition of system x_c_^−^. System x_c_^−^ - deficient mice, including transgenic mice with a deletion in exon one of the xCT gene (xCT^−/-^ mice, C57BL/6 J background) (Sato et al., 2005) and mice carrying a spontaneous subtle gray mutation which extends from intron 11 through exon 12 and results in truncated Slc7a11 mRNA (Sut mice, C3H/HeSnJ background) (Chintala et al., 2005), have been valuable tools to identify the involvement of system x_c_^−^ in disease progression in preclinical settings.

Using xCT^−/−^ mice, we demonstrated that system x_c_^−^ is the major source of extracellular glutamate in different brain regions (De Bundel et al., 2011; Massie et al., 2011). This glutamate can modulate the glutamatergic neurotransmission but when extracellular glutamate concentrations rise, e.g. in case of injury or neurological disorders (Mehta et al., 2013; Olloquequi et al., 2018), this glutamate can lower the threshold for glutamate toxicity (excitotoxicity) and thereby induce or further promote disease progression. Furthermore, system x_c_^−^ has been reported to drive neuroinflammation. Genetic xCT deletion shifts microglial cells towards a more anti-inflammatory, neuroprotective profile in a model for amyotrophic lateral sclerosis (Mesci et al., 2015) and attenuates the (neuro)inflammatory response after a systemic injection of a sublethal dose of lipopolysaccharide (LPS) (Albertini et al., 2018). Accordingly, xCT^−/−^ mice have been shown to be protected in models for several neurological disorders.

In the context of cancer, it is widely accepted that inhibition of system x_c_^−^ reduces cancer cell proliferation (Gout et al., 2001; Guo et al., 2011; Lewerenz et al., 2013; Dai et al., 2014), tissue invasion and metastasis (Sontheimer and Bridges, 2012; Lewerenz et al., 2013) as well as multidrug resistance (Lo et al., 2008; Sontheimer and Bridges, 2012; Lewerenz et al., 2013; Bhutia et al., 2015). While these features result from the inhibition of cystine uptake and consequently reduced glutathione synthesis in the cancer cells, also decreased glutamate release can be beneficial in some cancer types. In glioblastoma, glutamate released from the tumor by system x_c_^−^ is involved in peritumoral seizure development and favors cancer invasion by inducing peri-tumoral excitotoxic neuronal cell death (Sontheimer and Bridges, 2012). Moreover, in bone cancer, inhibition of system x_c_^−^ has been shown to reduce cancer-induced bone-pain due to decreased glutamate release (Ungard et al., 2014).

To allow translation of these findings to a clinical setting, pharmacological inhibition of the transporter is required. However, all candidate inhibitors that have been proposed, have their drawbacks and off-target effects. Cyclic and non-cyclic glutamate analogues, such as L-α-aminopimelate and (S)-4-carboxyphenylglycine, show cross-activity with glutamatergic receptors due to the structure similarity with glutamate (Lewerenz et al., 2013), and inhibitors such as sorafenib and erastin show neuronal toxicity (Dahlmanns et al., 2017). Moreover, toxic effects related to inhibition of tyrosine kinase by sorafenib (Granito et al., 2016) and nephrotoxicity due to cytotoxic effects of erastin on healthy renal cells (Fujiki et al., 2019; Yu et al., 2019) were demonstrated. Capsazepine blocks voltage-activated calcium channels (Docherty et al., 1997), vanilloid receptor and nicotinic acetylcholine receptors (Liu and Simon, 1997) and sulfasalazine (SAS) inhibits NFkB (Weber et al., 2000) and acts as an antagonist of the N-methyl-D-aspartate (NMDA) receptor (Ryu et al., 2003; Noh et al., 2006).

Despite its off-target effects (Weber et al., 2000; Ryu et al., 2003; Noh et al., 2006), SAS is the most frequently used molecule to test the potential of system x_c_^−^ as a drug target in both preclinical (Evonuk et al., 2015; Gout et al., 2001; Ma et al., 2015; Leclercq et al., 2019; Hu et al., 2020) and clinical studies (Shitara et al., 2017; Takeuchi et al., 2014). It is FDA- and EMA-approved and already on the market as an anti-inflammatory drug for decades, allowing a fast transfer to a clinical setting. While SAS-induced inhibition of system x_c_^−^ was effective in rodent models of different cancer types (Gout et al., 2001; Chung et al., 2005; Doxsee et al., 2007; Guo et al., 2011; Ma et al., 2015; Wada et al., 2018; Hu et al., 2020) as well as in models for epilepsy (Leclercq et al., 2019) and multiple sclerosis (MS) (Evonuk et al., 2015), Soria *et al.* recently reported *in vivo* myelin degeneration in the white matter of the spinal cord and *in vitro* oligodendrocyte-toxicity after SAS-induced chronic inhibition of system x_c_^−^ (Soria et al., 2016). On the contrary, blocking system x_c_^−^ during inflammation was suggested to prevent oligodendrocyte damage in white matter disorders (Domercq et al., 2007). Indeed, the use of SAS in the context of MS is debatable: some pre-clinical studies demonstrated beneficial effects of SAS treatment -including reduced demyelination (Evonuk et al., 2015; Prosiegel et al., 1990)- while others showed worsening of the clinical symptoms when the treatment was continued for a longer time (Correale et al., 1991; Noseworthy et al., 1998). Also in the context of glioblastoma, contradictory data have been published concerning the safety of SAS. Positive effects of SAS were seen in rodent models, without any signs of toxicity (Gout et al., 2001; Chung et al., 2005), yet two clinical trials with glioblastoma patients revealed severe side effects of SAS including neurological features as well as bone-marrow and hematological toxicity (mostly leukopenia and neutropenia) (Robe et al., 2009; Takeuchi et al., 2014).

For both ulcerative colitis and Crohn’s disease -the main indications of SAS- the pharmacological activity is driven by the anti-inflammatory metabolite mesalazine, which is formed after cleavage in the intestine. In animal models for these disorders, rather small doses of SAS (10–100 mg/kg) are administered orally (Radi et al., 2011; Shin et al., 2017; Soliman et al., 2019). However, to achieve inhibition of system x_c_^−^ SAS needs to reach the plasma and the target organ in its intact form. As such, higher doses (150–320 mg/kg twice a day) and other routes of administration (mostly intraperitoneal (i.p.) injections) have been used (Evonuk et al., 2015; Gout et al., 2001; Chung et al., 2005; Lo et al., 2010). While orally taken SAS is overall well-tolerated in patients with ulcerative colitis and Crohn’s disease (Rains et al., 1995), it is possible that the increased systemic concentrations of intact SAS, result in toxic effects of SAS itself or its metabolite sulphapyridine (Zheng et al., 1993).

In this study, we aimed to identify the (adverse) effects of SAS that are mediated by inhibition of system x_c_^−^. To do so, we chronically i. p. administered two doses of SAS that are commonly used to achieve inhibition of system x_c_^−^ in preclinical studies -i.e. 160 mg/kg twice a day or 320 mg/kg once a day (Evonuk et al., 2015; Gout et al., 2001; Chung et al., 2005; Soria et al., 2016)- to xCT^−/-^ mice and their wildtype littermates (xCT^+/+^ mice), and studied the welfare of the mice as well as different behavioral outcomes. Given inconsistent reports on the use of SAS in disorders characterized by deficits in the spinal cord such as MS, we further focused on the involvement of system x_c_^−^ in possible toxic effects of chronic SAS treatment on the spinal cord.

## Material and Methods

### Animals

Six-month-old male xCT^−/-^ and xCT^+/+^ littermates were used. These mice are high-generation descendants (more than 15 back-crosses on a C57BL/6 J background) of the strain originally described by Sato et al. (2005), and are bred in a heterozygous colony. Mice were genotyped by a PCR amplification on DNA extracted from ear punches, using the REDExtract-N-Amp Tissue PCR kit (Sigma-Aldrich) and the following primers: 5”‐GATGCCCTTCAGCTCGATGCGGTTCACCAG‐3“(GFPR3); 5”‐CAGAGCAGCCCTAAGGCACTTTCC‐3“(mxCT5” flankF6); 5”‐CCG​ATG​ACG​CTG​CCG​ATG​A TGATGG‐3”(mxCT [Dr.4]R8). Mice were group-housed under standardized conditions (20–24°C, 10/14 h dark/light cycle, 45–65% humidity) with free access to water and food. Animal experiments were approved by the Ethical Committee for Animal Experiments of the Vrije Universiteit Brussel and carried out according to the national guidelines on animal experimentation. All efforts were made to minimize animal suffering.

### Experimental design

**FIGURE 1:**
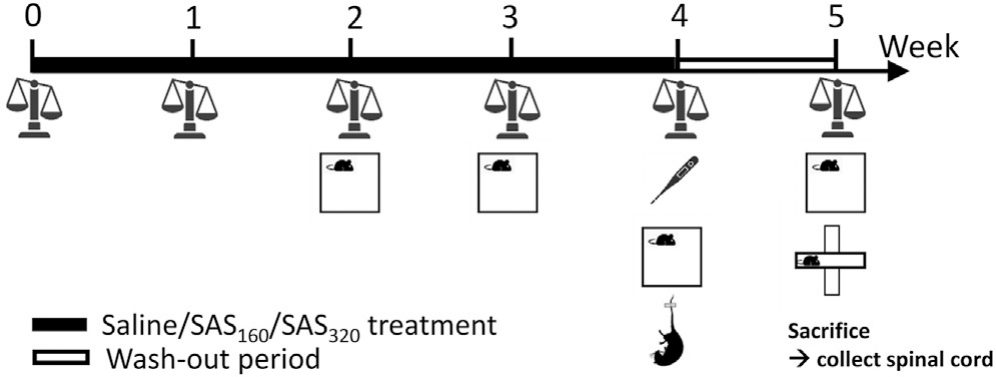
Graphical illustration of the experimental design. Over a period of four weeks, mice were treated with SAS (or saline), followed by a wash-out period of one week. Every week, the body weight of the mice was measured and starting from the second week of treatment, effects of SAS on locomotor function and anxiety-like behavior were analyzed using the open field test (OF). After four weeks of treatment, we evaluated the effect of SAS on body temperature as well as depressive-like behavior using the mouse tail suspension test. At the end of the wash-out period, long-lasting effects of SAS on body weight, locomotor function and anxiety-like behavior (using the OF as well as the elevated plus maze test) were studied. After behavioral testing, mice were sacrificed and the spinal cord was harvested for further analysis.

A fresh 40 mM SAS (2-hydroxy-5-[[4-(pyridin-2-ylsulfamoyl)phenyl]diazenyl]benzoic acid; Sigma-Aldrich) solution was prepared daily by dissolving the powder in a small volume of NaOH 0.1 M. pH was adjusted to 7.4 and saline (0.9% NaCl, B. Braun Vet Care) added to reach the desired concentration of SAS. Mice were randomly assigned to the different treatment groups (160 mg/kg of SAS twice a day, 320 mg/kg of SAS once a day or saline twice a day). Over a period of four weeks, mice were i. p. injected with SAS (or saline) at 10:00 a.m. (all mice) and 5:00 p.m. (mice treated with 160 mg/kg of SAS and saline). As indicated in Figure 1, the body weight of each animal was measured every week to adjust the dose of SAS. Starting from the second week of treatment, effects of SAS on locomotor function and anxiety-like behavior were analyzed using the open field (OF) test, 2 h after the first injection of the day (12:00 a.m.). After four weeks of treatment, we evaluated the effect of SAS on body temperature (immediately after the first injection of the day), as well as depressive-like behavior using the mouse tail suspension test (MTS, 1 h after the OF). Next, we included a wash-out period of one week during which the mice did not receive SAS or saline injections and studied the long-lasting effects of SAS on body weight, locomotor function and anxiety-like behavior (using the OF as well as the elevated plus maze (EPM) test). After behavioral testing, mice were sacrificed by cervical dislocation and spinal cord was harvested and post-fixed in 4% paraformaldehyde for 72 h. Of note, for each behavioral test, mice were acclimatized to the testing room at least 1 h prior to assessment and all analyses were performed by a researcher blinded for treatment and genotype.

### Open-Field Test

In the OF, mice were placed in a corner of a square box (60 × 60 × 60 cm) with surrounding black opaque walls that prevent observation of visual cues outside the arena (Bentea et al., 2015). The center of the arena was defined as the central 40 × 40 cm zone. The illuminance in the arena was 150 lux in the center and 30 lux in the corners. Mice were allowed to explore the arena for 5 min and the experiment was video-recorded. The total distance travelled (parameter for spontaneous locomotion and explorative behavior) as well as the cumulative duration spent in the center zone (parameter for anxiety-like behavior) were analyzed using an automated video tracking system (Ethovision software, Noldus).

### Mouse Tail Suspension Test

For the MTS, mice were suspended by the tip of their tail for 5 min to induce an inescapable situation (Bentea et al., 2015). The mice were video-recorded and the time of immobility, which is considered as a parameter for depressive-like behavior (Steru et al., 1985), was measured manually. Mice that climbed their tail were excluded from the analysis as they learned that escape is possible, thereby invalidating immobility time as measure for depressive-like behavior (Cryan and Mombereau, 2004).

### Elevated Plus Maze Test

In the EPM, mice were placed in the corner of a closed arm of an elevated (37 cm from the ground) cross-shaped maze consisting of two open and two enclosed arms (32.5 cm length × 6 cm width × 17 cm height), with a center area of 6 × 6 cm (Rodgers et al., 1995). The illuminance in the center was 150 lux. Mice were allowed to explore the maze for 5 min and the experiment was video-recorded. The time spent in the open arms (parameter for anxiety-like behavior), the total distance travelled and velocity (parameters to evaluate locomotor functions) were analyzed using an automated video tracking system (Ethovision software).

### Body Temperature

The body temperature of the mice was recorded before as well as 10, 60 and 120 min after i. p. injection with SAS or saline, using a rectal probe (RET-3, ADinstruments) connected to a thermometer (Testo 935, Testo). Mice were restrained, but not anesthetized, to perform the procedure.

### Histology

The post-fixed spinal cords were embedded in paraffin, sliced into 10 µm sections using a microtome, mounted on glass slides and dried overnight at 42°C. Slides containing sections of the cervical region of the spinal cord were soaked in Eriochrome Cyanine (Sigma-Aldrich) for 30 min and after rinsing with deionized water, slides were processed in a bath of differentiator (ammonium hydroxide). Counterstaining was performed with Neutral Red (Thermo Scientific). Stained sections were examined using a Leica 2450 microscope (Leica Microsystems Gmbh).

### Immunohistochemistry

Neurons, astrocytes, oligodendrocytes, microglia and myelin were labeled in paraffin sections of the cervical region of the spinal cord, using respectively anti-NeuN, anti-GFAP, anti-p25α, anti-Iba1, anti-myelin basic protein (MBP) and anti-myelin-associated glycoprotein (MAG) antibodies (see Table 1). Paraffin sections were dewaxed, rehydrated and epitope retrieval was performed with citrate buffer (pH 6) at 100°C for 10 min. Endogenous peroxidase activity was eliminated by incubation with 3% H_2_O_2_ for 10 min. Sections were placed in a bath of 0.1 M glycine for 3 min, followed by a 30 min blocking step in 5% goat or horse serum diluted in Tris-buffered saline (TBS; see Table 1) to avoid non-specific binding of antibodies. Sections were incubated with primary antibodies diluted in 1% normal goat or horse serum (in TBS) overnight at 4°C (see Table 1). The next day, after rinsing, sections were incubated for 1 h in biotinylated secondary antibodies (1:300; ABC Kit, Vectastain) at room temperature, followed by peroxidase-bound streptavidin (1:200, ABC Kit) for 45 min. Immunoreactivity was revealed using 3,3 di-amino-benzidine (Dako), counterstained using hemalum before dehydration and observed under an Olympus BX63 microscope (Olympus lifescience). The number of NeuN^+^ and p25α^+^ cells was assessed quantitatively in the grey matter, using the Cell Sens software. The same software was used to quantify the MAG immunoreactivity in the white matter. MBP immunoreactivity was manually scored for the presence of myelin abnormalities: normal (0), vacuolization or myelin disorganization (1) or myelin loss (2). Astrogliosis was evaluated in the gray matter by manually scoring sections stained for GFAP as follows: normal (0), presence of slight (1), mild (2) or severe astrogliosis (3). Finally, Iba-1 positive microglia in the gray matter were classified into different activation states (type A-D) based on morphology, as described before (Bouchat et al., 2017).

**TABLE 1:** Primary antibodies used for immunostaining.

Primary antibodies [species]	Dilution	Supplier	References	Blocking solution
Anti-Iba1 [PR]	1:1000	Wako chemicals	019-9741	NGS
Anti-p25α [PR]	1:1000	Sigma-Aldrich	PA036576	NGS
Anti-GFAP [MM]	1:10 000	Sigma-Aldrich	GA5 clone, G3893	NHS
Anti-MAG [MM]	1:5000	Abcam	ab89780	NHS
Anti-MBP [PR]	1:500	Abcam	ab40390	NGS
Anti- NeuN [PR]	1:1000	Cell Signaling	D3S3I	NGS

PR: polyclonal rabbit; MM: mouse monoclonal; NGS: normal goat serum; NHS: normal horse serum.

### Statistics

Data are presented as mean ± standard error of the mean (SEM). For all analyses, we evaluated the effect of SAS treatment in each genotype separately. For data including a time effect, a two-way ANOVA followed by a Sidak's multiple comparisons test (MCT) comparing each dose of SAS to the saline group or a Wilcoxon matched-pairs signed rank test (Wilcoxon MSRT) was used. For data on one timepoint, a Kruskal-Wallis (KW) test was performed followed by a Dunn’s MCT comparing each dose of SAS to the saline group. Categorical data was analyzed using a Fisher's exact test and survival curves were analyzed using a Log-rank test. All analyses were performed in GraphPad Prism eight and the α-value was set at 0.05.

## Results

### Chronic Sulfasalazine Treatment Induces Mortality and Weight Loss in a xCT-independent Manner

**FIGURE 2:**
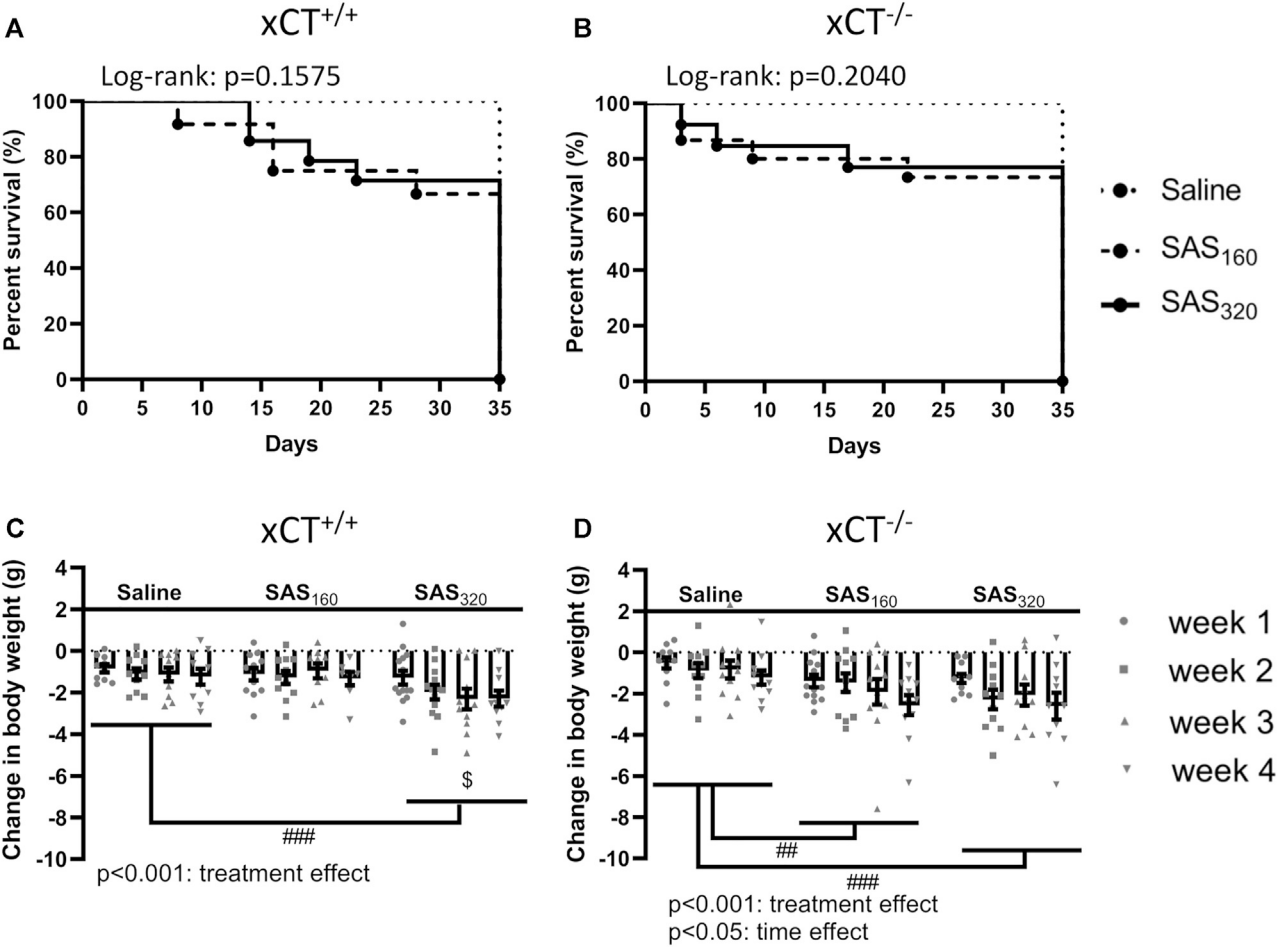
The effect of chronic sulfasalazine (SAS) treatment on survival and body weight. Survival curves of xCT^+/+^ (*n* = 10–14 mice/group) **(A)** and xCT^−/−^ mice (*n* = 11–15 mice/group) **(B)** were determined using a Kaplan-Meier curve and analyzed with a Log-rank test. Body weight of xCT^+/+^ (*n* = 10–14 mice/group at the start of the experiment, *n* = 8–11 mice/group after four weeks of treatment) **(C)** and xCT^−/−^ mice (*n* = 11–15 mice/group at the start of the experiment, *n* = 10–11 mice/group after four weeks of treatment) **(D)** was recorded weekly and plotted as the change compared to baseline (i.e. the weight at the start of the treatment). Data are presented as mean ± SEM and analyzed using a two-way ANOVA followed by a Sidak's multiple comparisons test for each dose of SAS compared to saline. ^##^p < 0.01, ^###^p < 0.001: for treatment effect over all timepoints; ^$^p < 0.05: for treatment effect at one specific timepoint.

We started the experiment with *n* = 10 xCT^+/+^ mice and *n* = 11 xCT^−/−^ mice in the saline group, *n* = 12 xCT^+/+^ mice and *n* = 15 xCT^−/−^ mice in the SAS 160 mg/kg group, and *n* = 14 xCT^+/+^ mice and *n* = 13 xCT^−/−^ mice in the SAS 320 mg/kg group. Over the four weeks of SAS treatment, four mice of each genotype treated with 160 mg/kg of SAS as well as four xCT^+/+^ and three xCT^−/−^ mice treated with 320 mg/kg of SAS died unexpectedly without any clear preceding sickness behavior. None of the mice injected with saline died during the experiment (Figures 2A,B).

While all mice lost weight starting from the first week of treatment, this was more pronounced in SAS-treated mice (Figures 2C,D; two-way ANOVA, treatment effect: xCT^+/+^ mice F_(2,116)_ = 8.197, *p* = 0.0005; xCT^−/−^mice F_(2,122)_ = 8.176, *p* = 0.0005). Injections with both saline and 160 mg/kg of SAS induced the same degree of weight loss in xCT^+/+^ mice, which was stable over the four weeks of treatment (Figure 2C; Sidak’s MCT: *p* = 0.9251), whereas the weight loss was more pronounced in xCT^+/+^ mice treated with 320 mg/kg of SAS compared to saline-injected mice (Figure 2C; Sidak’s MCT: *p* = 0.0008). In contrast, all xCT^−/−^ mice treated with SAS lost more weight compared to saline-treated mice of the same genotype (Figure 2D; Sidak’s MCT, saline vs. SAS_160_: *p* = 0.0039, saline vs. SAS_320_: *p* = 0.0005). Moreover, weight loss increased over time in all groups of xCT^−/−^ mice, with the most pronounced weight loss after four weeks of daily injections (Figure 2D; two-way ANOVA, time effect: F_(3,122)_ = 2.982, *p* = 0.0340; Sidak’s MCT, w1 vs. w4: *p* = 0.0203).

### Chronic sulfasalazine treatment decreases the total distance travelled in the open field arena but does not induce anxiety- or depressive-like behavior

**FIGURE 3:**
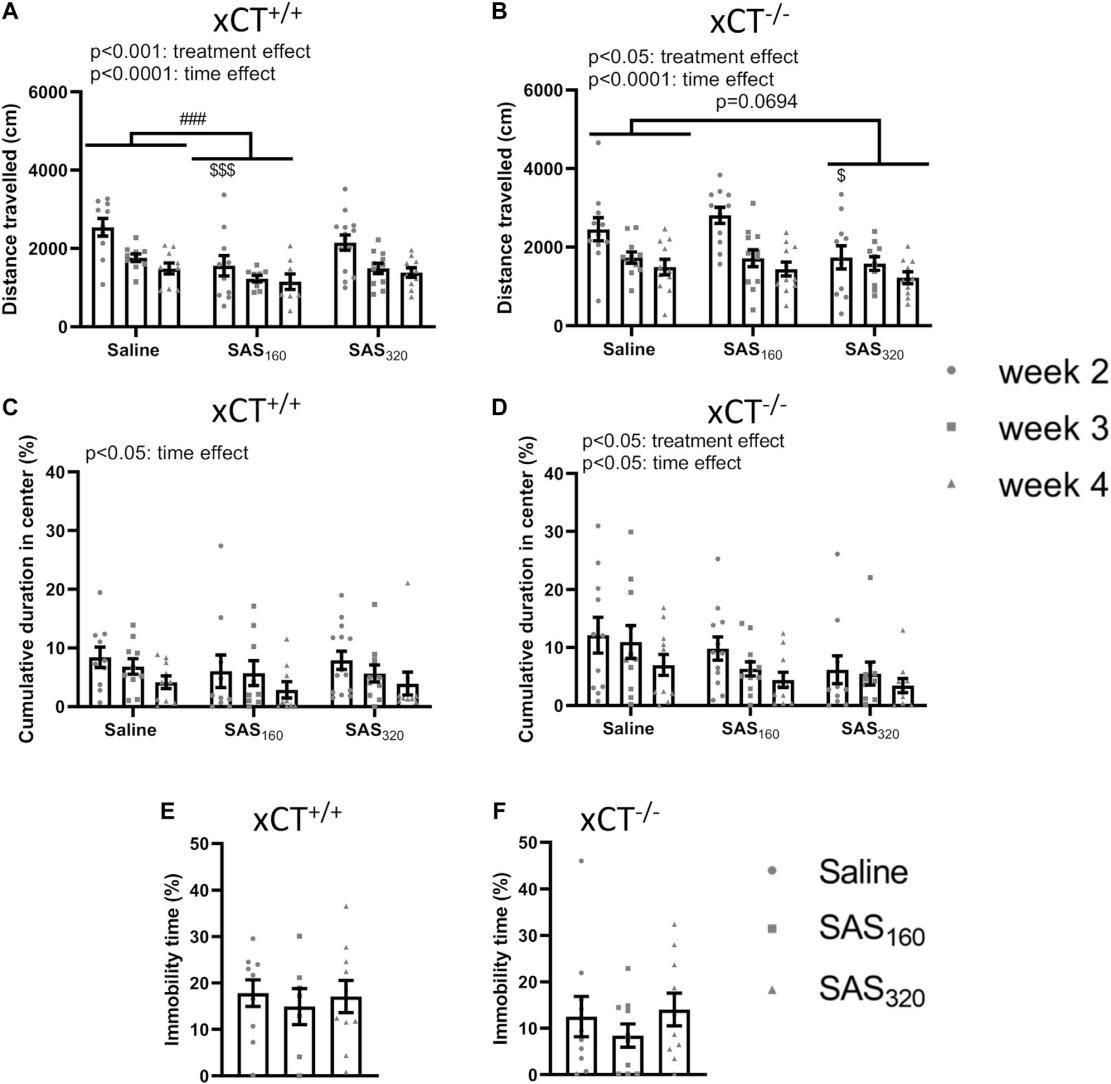
The effect of chronic sulfasalazine (SAS) treatment on locomotor function as well as anxiety- and depressive-like behavior. The total distance travelled in the open field (OF) test was used to measure locomotor function of xCT^+/+^ (*n* = 10–14 mice/group at the start of the experiment, *n* = 8–11 mice/group after four weeks of treatment) **(A)** and xCT^−/−^ mice (*n* = 11–12 mice/group at the start of the experiment, *n* = 10–11 mice/group after four weeks of treatment) **(B)**, starting from the second week of treatment. The cumulative duration spent in the center of the OF arena was plotted to study anxiety-like behavior **(C,D)**. The immobility-time in the mouse tail suspension test (MTS) performed after four weeks of treatment was assessed as a parameter for depressive-like behavior (*n* = 7–10 xCT^+/+^mice/group **(E)**; *n* = 10 xCT^−/−^ mice/group **(F)**). Data are presented as mean ± SEM and analyzed using a two-way ANOVA followed by Sidak's multiple comparisons for each dose of SAS compared to saline for the OF: ^##^ *p* < 0.01, ^###^ *p* < 0.001: for treatment effect over all timepoints; ^$^ *p* < 0.05, ^$$$^ *p* < 0.001: for treatment effect at one specific timepoint. MTS data were analyzed using a Kruskal-Wallis test.

Starting from week 2 of treatment, locomotor function of the mice was analyzed using the OF (Figures 3A,B). All xCT^+/+^ mice covered a longer distance in the OF arena the first time they were introduced to this test, compared to all other timepoints (Figure 3A; two-way ANOVA, time effect: F_(2,842)_ = 14.85, *p* < 0.0001; Sidak’s MCT, w2 vs. w3: *p* = 0.0003, w2 vs. w4: *p* < 0.0001). The same time-effects were seen in xCT^−/−^ mice (Figure 3B; two-way ANOVA: F_(2,902)_ = 15.60, *p* < 0.0001; Sidak’s MCT, w2 vs. w3: *p* = 0.0008, w2 vs. w4: *p* < 0.0001). In addition, SAS-treated xCT^+/+^ mice traveled less over the entire period of testing compared to saline-injected mice of the same genotype (Figure 3A; two-way ANOVA, treatment effect: F_(2,84)_ = 8.397, *p* = 0.0005). This effect was driven by the mice treated with 160 mg/kg of SAS (Sidak’s MCT, saline vs. SAS_160_ over the entire period of testing: *p* = 0.0002) and it is most pronounced in the first test trial (Sidak’s MCT, saline vs. SAS_160_ at w2: *p* = 0.0003). Also in xCT^−/−^ mice, SAS treatment significantly affected the distance walked in the OF arena (Figure 3B; two-way ANOVA, treatment effect: F_(2,90)_ = 4.043, *p* = 0.0208). In contrast to the xCT^+/+^ mice, this parameter was not significantly altered between xCT^−/−^ mice treated with saline or 160 mg/kg of SAS, whereas there was a trend towards a decrease in xCT^−/−^ mice treated with 320 mg/kg of SAS compared to the saline-treated ones (Figure 3B; Sidak’s MCT, saline vs. SAS_320_ over the entire period of testing: *p* = 0.0694). In line with the treatment effect of 160 mg/kg of SAS in xCT^+/+^ mice, this effect is mostly seen at week 2 of the treatment (Sidak’s MCT, saline vs. SAS_320_ at w2: *p* = 0.0398).

The cumulative duration in the center of the OF arena was-in agreement with the data on the total distance travelled- the highest after two weeks of treatment (the first time they explore the OF arena) in all tested groups (Figures 3C,D). The time in the center gradually decreased over the treatment period in all groups of xCT^+/+^ and xCT^−/−^ mice, resulting in a significant difference between the performance on week 2 compared to week 4 (Figure 3C; two-way ANOVA, xCT^+/+^ mice: time effect F_(2,84)_ = 3.489, *p* = 0.0350; Sidak’s MCT, w2 vs. w4: *p* = 0.0307; xCT^−/−^ mice: time effect F_(2,90)_ = 3.401, *p* = 0.0377; Sidak’s MCT, w2 vs. w4: *p* = 0.0326). While we were unable to show a treatment effect in xCT^+/+^ mice (Figure 3C; two-way ANOVA: F_(2.84)_ = 0.5784, *p* = 0.5630), SAS treatment significantly decreased the time spent in the center of the OF arena in xCT^−/−^ mice (Figure 3D; two-way ANOVA, treatment effect F_(2,90)_ = 4.283, *p* = 0.0167). The latter effect is driven by the mice treated with 320 mg/kg of SAS (Sidak’s MCT, saline vs. SAS_320_ over the entire period of testing: *p* = 0.0099). Finally, there were no effects of four weeks of SAS treatment on the immobility time in the MTS test -which was used to measure depressive-like behavior-independent of the dose of SAS in both xCT^+/+^ (Figure 3E; KW test: *p* = 0.8149) and xCT^−/−^ mice (Figure 3F; KW test: *p* = 0.5502).

### Chronic Sulfasalazine Treatment Influences Body Temperature via xCT-Independent Mechanisms

**FIGURE 4:**
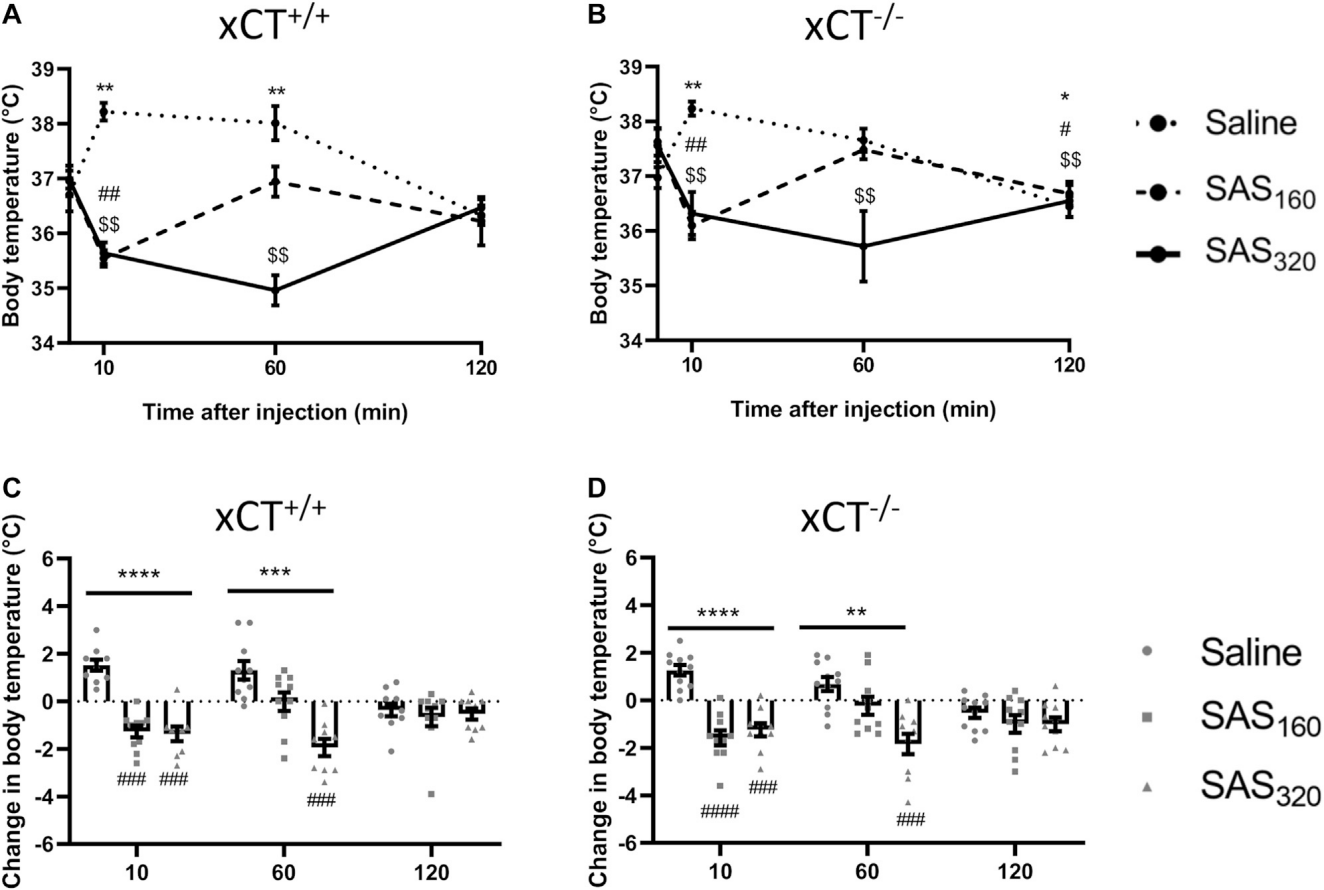
The effect of chronic sulfasalazine (SAS) treatment on changes in body temperature after injection. After four weeks of treatment, body temperature was measured 10, 60 and 120 min after SAS injection and plotted as absolute values **(A,B)** or as the difference compared to the body temperature before injection **(C,D)** (*n* = 9–10 x CT^+/+^ mice/group **(A,C)**; *n* = 10–11 xCT^−/−^ mice/group **(B,D)**). Data are presented as mean ± SEM and analyzed using a Wilcoxon matched-pairs signed rank test for each treatment paradigm at the different timepoints compared to baseline (A–B): saline: *p < 0.05, **p < 0.01; SAS_160_: ^#^ *p* < 0.05, ^##^ *p* < 0.01; SAS_320_: ^$$^ *p* < 0.01. A Kruskal-Wallis test (C-D; **p < 0.01, ***p < 0.001, ****p < 0.0001) followed by Dunn’s multiple comparison was used to compare each dose of SAS to saline on each timepoint (^###^ *p* < 0.001, ^####^ *p* < 0.0001).

Immediately after SAS injection some of the mice did not show their normal behavior but were lying on their abdomen and seemed to be panting. As this behavior has been described to be related to thermoregulation (McDonough et al., 2020), we evaluated the effect of SAS injection on the body temperature of the mice. As presented in Figure 4A, saline-treated xCT^+/+^ mice showed a normal stress-induced hyperthermic reaction after an i. p. injection (Olivier et al., 2003), with an initial 2°C increase of their body temperature, which normalizes after 120 min (Wilcoxon MSRT compared to baseline, 10 min: *p* = 0.0020, 60 min: *p* = 0.0059, 120 min: *p* = 0.2598). However, 10 min after injection, the body temperature of all SAS-treated xCT^+/+^ mice was decreased compared to baseline (Figure 4A; Wilcoxon MSRT compared to baseline, SAS_160_: *p* = 0.0039, SAS_320_: *p* = 0.0039). As such, the change in body temperature as a result of the i. p. injection was significantly different between both groups of SAS-treated xCT^+/+^ mice compared to saline-injected mice of the same genotype (Figure 4C; KW test: *p* < 0.0001; Dunn’s MTC, saline vs. SAS_160_: *p* = 0.0010, saline vs. SAS_320_: *p* = 0.0001). The change in body temperature of xCT^+/+^ mice treated with 320 mg/kg of SAS was still significantly different from saline-treated xCT^+/+^ mice 60 min after injection (Figure 4C; KW test: *p* = 0.0003; Dunn’s MCT: *p* = 0.0001), contrary to the 160 mg/kg group (Dunn’s MCT: *p* = 0.2417). The same response was seen in xCT^−/−^ mice, with an increased body temperature in saline-treated xCT^−/−^ mice 10 min after injection (Figure 4B; Wilcoxon MSRT compared to baseline; saline: *p* = 0.0020), while both doses of SAS induced hypothermia (Figure 4B; Wilcoxon MSRT compared to baseline, SAS_160_: *p* = 0.0020, SAS_320_ *p* = 0.0039), resulting in a significant difference in the injection-induced change in body temperature between SAS- and saline-treated mice (Figure 4D; KW test: *p* < 0.0001; Dunn’s MTC, saline vs. SAS_160_: *p* < 0.0001, saline vs. SAS_320_: *p* = 0.0010). For the xCT^−/−^ mice treated with 320 mg/kg of SAS, this effect was still present 60 min after injection (Figure 4D; KW test *p* = 0.0011; Dunn’s MTC: *p* = 0.0004), in contrast to the ones treated with 160 mg/kg of SAS (Dunn’s MTC: *p* = 0.2065). Taken together, all SAS-induced effects on body temperature were seen in both genotypes and only temporary as at 120 min after injection no difference in the change in body temperature was seen between SAS- and saline-injected mice, independent of genotype (Figures 4C,D; KW test, xCT^+/+^ mice: *p* = 0.7696, xCT^−/−^ mice: *p* = 0.4688).

### Chronic Sulfasalazine Treatment Does Not Induce Long Term-Effects on Behavior

**FIGURE 5:**
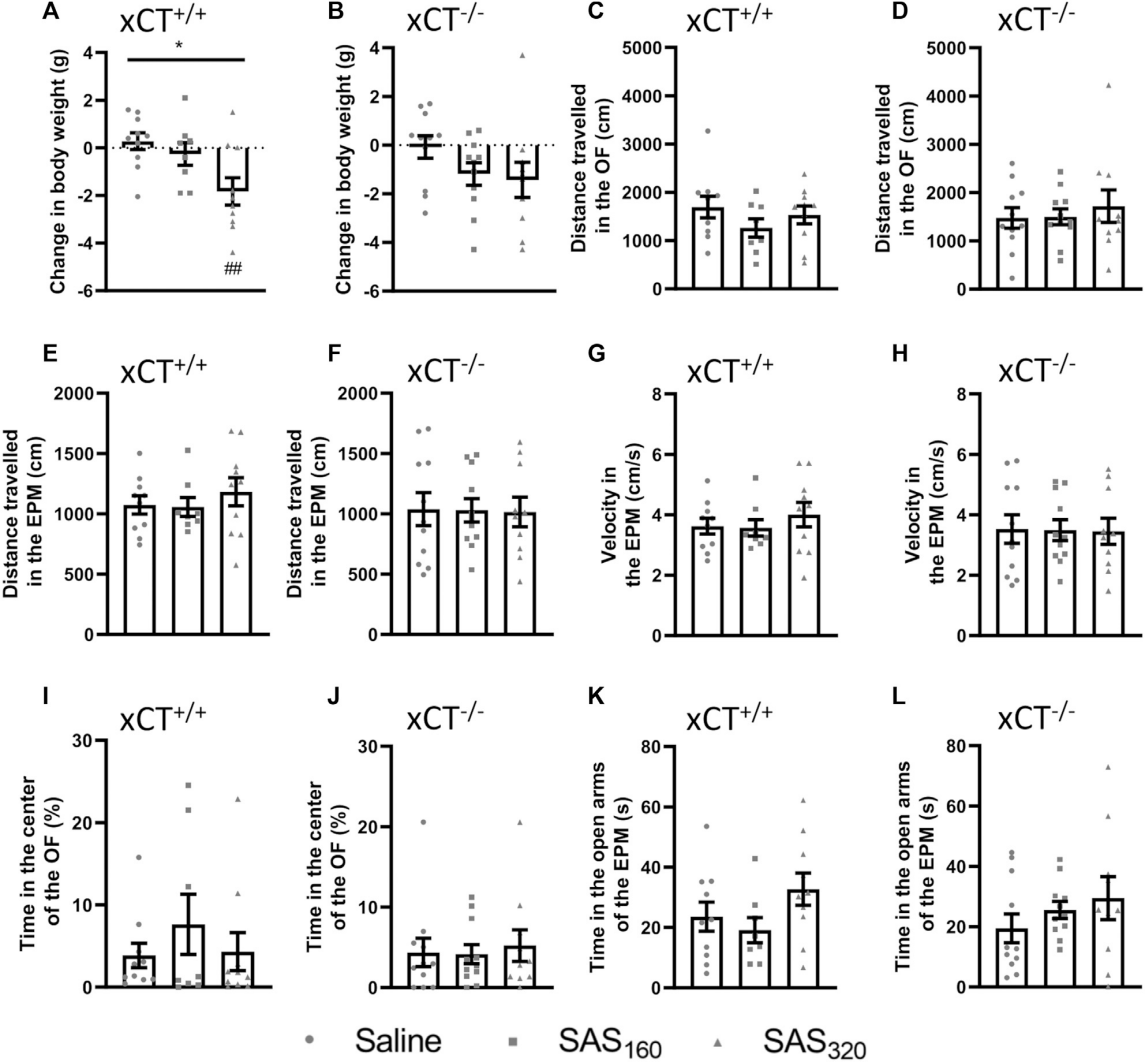
Long-term effects of chronic sulfasalazine (SAS) treatment. After a wash-out period of one week, body weight of xCT^+/+^ **(A)** and xCT^−/−^ mice **(B)** was evaluated and plotted as the change compared to baseline (i.e. bodyweight at the start of the treatment). Distance travelled in the open field (OF) arena **(C,D)** or the elevated plus maze (EPM) **(E,F)**, and velocity while exploring the EPM **(G,H)** were evaluated and represent long-term effects on locomotor function. Time spent in the center of the OF **(I, J)** or in the open arms of the EPM **(K,L)** was used as a parameter for anxiety-like behavior. For all tests, *n* = 8–10 xCT^+/+^ mice/group and *n* = 10–11 xCT^−/−^ mice/group were used. Data are presented as mean ± SEM and analyzed using a Kruskal-Wallis test (*p < 0.05) followed by Dunn’s multiple comparisons for each dose of SAS compared to saline (^##^ *p* < 0.01).

After a wash-out period of one week, we still detected an effect of SAS treatment on the body weight of xCT^+/+^ mice (Figure 5A; KW test: *p* = 0.0160). This effect was driven by the group receiving 320 mg/kg SAS. The change in bodyweight compared to baseline was significantly different in the xCT^+/+^ mice treated with 320 mg/kg of SAS compared to saline-treated xCT^+/+^ mice at this timepoint (Dunn’s MTC: *p* = 0.0097), while the effect of 160 mg/kg of SAS on body weight was faded out (Dunn’s MTC: *p* = 0.8347). Similarly, after the wash-out period the body weight of xCT^−/−^ mice treated with SAS is lower compared to the ones treated with saline, but this effect was not statistically significant (Figure 5B; KW test: *p* = 0.0907).

No long-term behavioral effects of SAS treatment were detected (Figure 5C–L). In xCT^+/+^ as well as xCT^−/−^ mice, the distance walked was unaffected by chronic SAS treatment in both the OF (Figures 5C,D; KW test, xCT^+/+^: *p* = 0.4502, xCT^−/−^: *p* = 0.9664) and EPM (Figures 5E,F; KW test, xCT^+/+^: *p* = 0.6306, xCT^−/−^: *p* = 0.9899). Also the velocity in the EPM paradigm was unaffected by chronic SAS treatment in mice of both genotypes (Figures 5G,H; KW test, xCT^+/+^ mice: *p* = 0.6306, xCT^−/−^ mice: *p* = 0.9936). Furthermore, independent of the genotype of the mice and regardless of the dose of SAS, no effect could be detected on the cumulative duration in the center of the OF (Figures 5I,J; KW test, xCT^+/+^ mice: *p* = 0.7243, xCT^−/−^ mice: *p* = 0.9563), neither on the time spent in the open arms of the EPM (Figures 5K,L; KW test, xCT^+/+^ mice: *p* = 0.2231, xCT^−/−^ mice: *p* = 0.4807). All together, these results demonstrate that chronic SAS treatment did not induce long-term changes in motor function or anxiety-like behavior.

### Chronic Sulfasalazine Treatment Does Neither Induce Neuronal Loss nor Changes in Myelin or Glial Cells in the Spinal Cord

**FIGURE 6:**
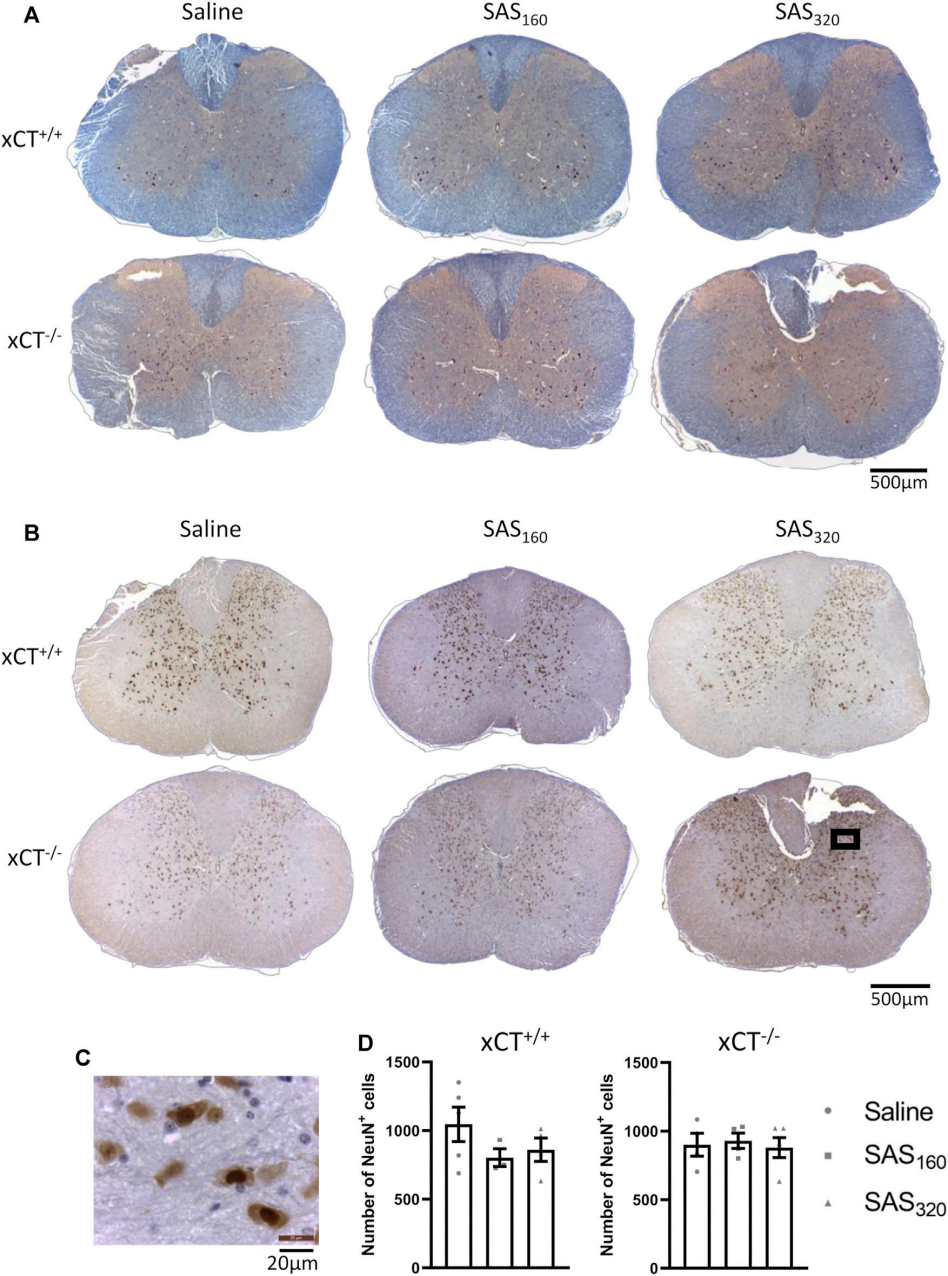
Histological examination of the spinal cord of mice after chronic sulfasalazine (SAS) treatment. Representative photomicrographs of the eriochrome cyanine/neutral red staining **(A)** as well as the NeuN immunohistochemistry **(B)**. A higher magnification picture of the boxed area in (B) is shown in **(C)**. NeuN^+^ cells were quantified using the Cell Sens software **(D)**. One slice of *n* = 3–5 mice/group was used, data are presented as mean ± SEM and analyzed using a Kruskal-Wallis test followed by Dunn’s multiple comparisons for each dose of SAS compared to saline.

Four weeks of treatment with either 160 mg/kg or 320 mg/kg of SAS did not induce any histological abnormalities in the spinal cord of xCT^+/+^ or xCT^−/−^ mice (Figure 6A). Also NeuN quantification did not show any significant effect of SAS treatment on the number of neurons present in the gray matter of the spinal cord (Figures 6B–D; KW test, xCT^+/+^: *p* = 0.4600, xCT^−/−^: *p* = 0.9523).

**FIGURE 7:**
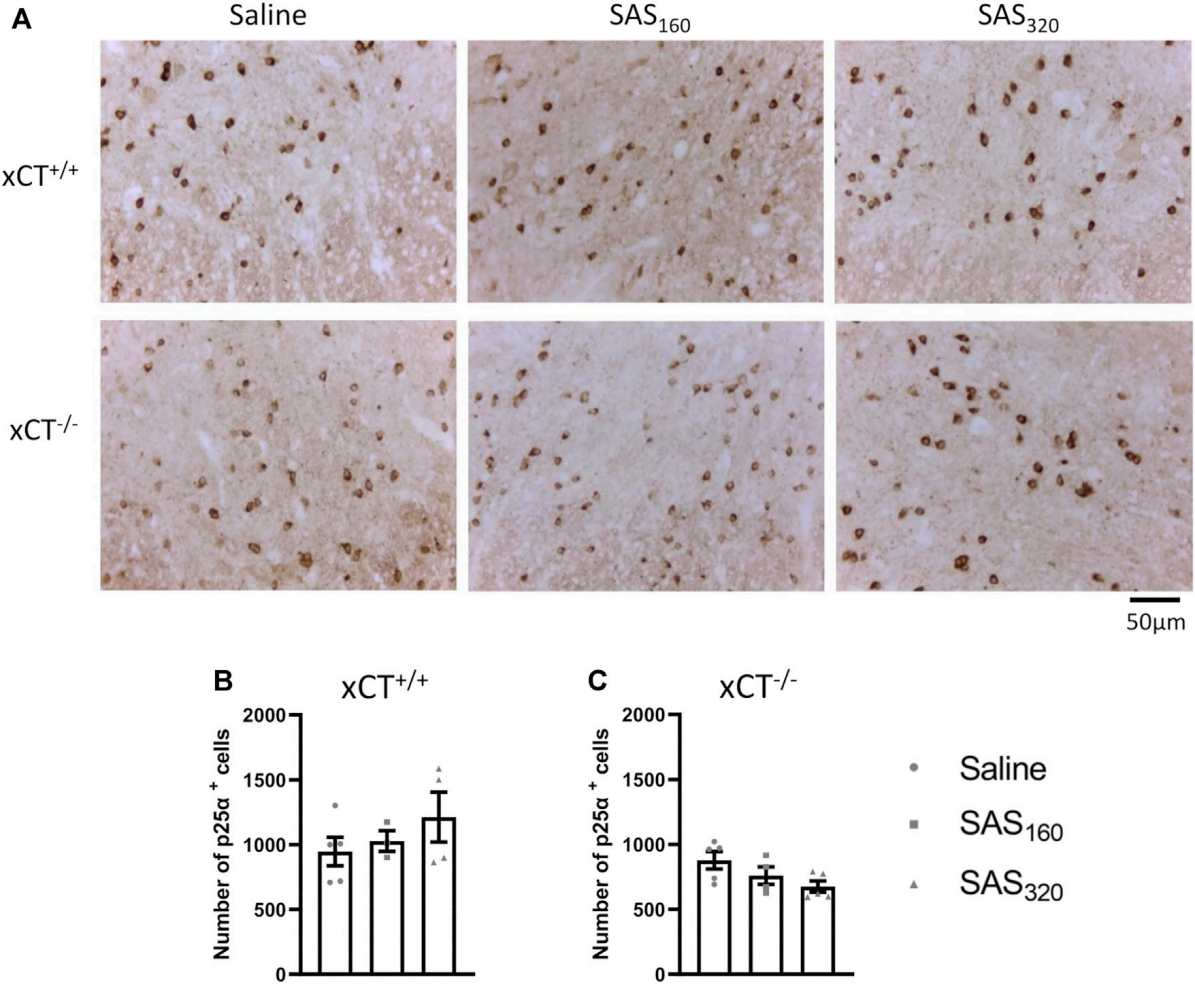
Effects of chronic sulfasalazine (SAS) treatment on oligodendrocytes in the spinal cord. Representative photomicrographs **(A)** and quantification **(B,C)** of p25α immunostaining to analyze oligodendrocytes. One slice of *n* = 3–5 mice/group was used, data are presented as mean ± SEM and analyzed using a Kruskal-Wallis test followed by Dunn’s multiple comparisons for each dose of SAS compared to saline.

**FIGURE 8:**
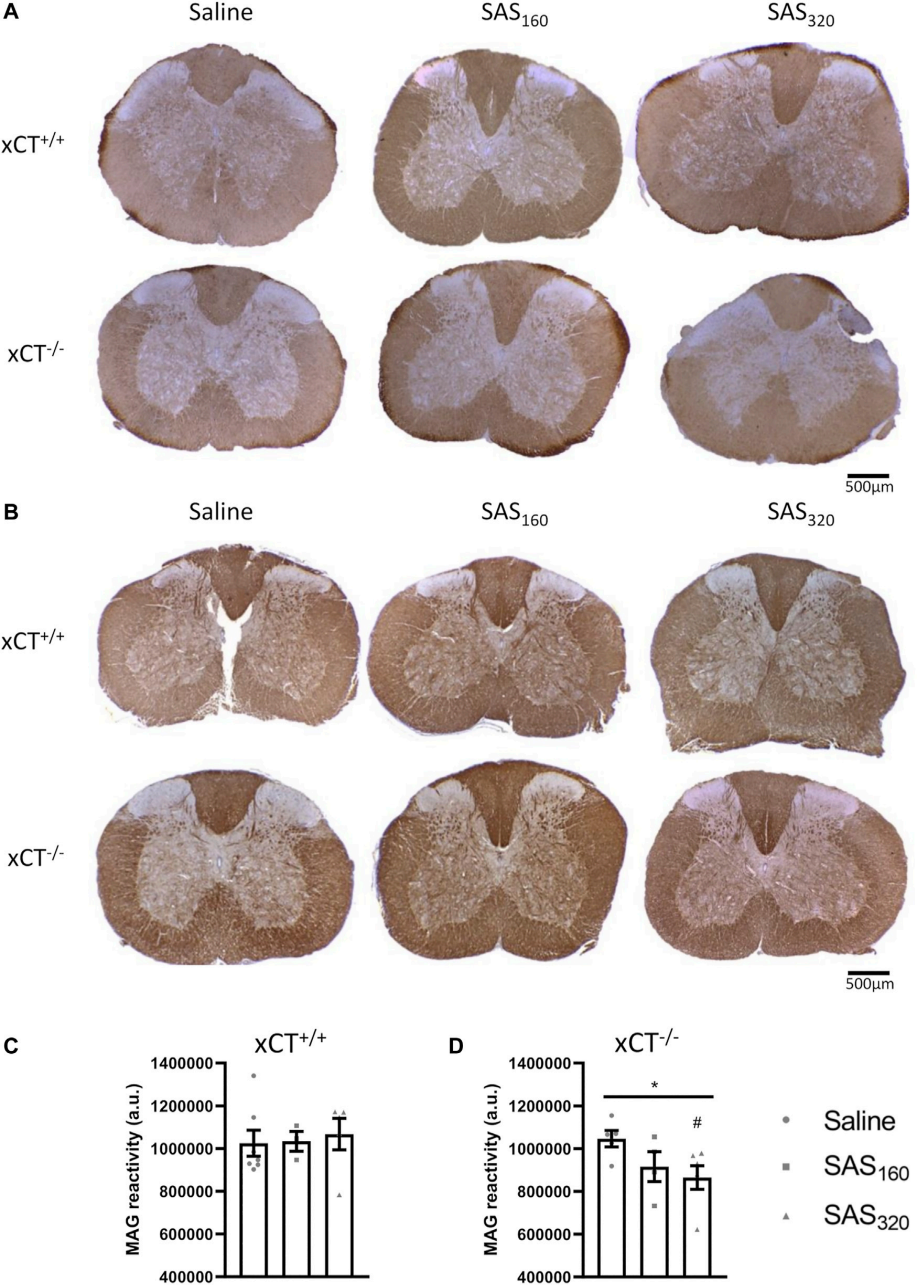
Effects of chronic sulfasalazine (SAS) treatment on myelin in the spinal cord. Representative photomicrographs of myelin basic protein (MBP) **(A)** and myelin-associated glycoprotein (MAG) immunostaining **(B)** as well as quantification of the MAG staining **(C,D)**. One slice of *n* = 3–7 mice/group was used, data are presented as mean ± SEM and analyzed using a Kruskal-Wallis test (*p < 0.05) followed by Dunn’s multiple comparisons for each dose of SAS compared to saline (^#^ *p* < 0.05).

Immunohistochemical analysis of p25α showed no difference in the number of oligodendrocytes in the gray matter of the spinal cord between saline and SAS-treated xCT^+/+^ (Figures 7A,B; KW test: *p* = 0.6024) or xCT^−/−^ mice (Figures 7A,C; KW test: *p* = 0.0889). All scores of the MBP staining were 0, indicating absence of vacuolization, myelin disorganization or myelin loss, regardless of the treatment or the genotype of the mice (Figure 8A). Furthermore, we quantified MAG immunoreactivity in the white matter as loss of the minor myelin proteins has been described as an early and sensitive biomarker for myelin degeneration in MS demyelinating lesions (Popescu and Lucchinetti, 2012). MAG stainings did not reveal any effect of SAS on the quality of the myelin sheet surrounding axons of xCT^+/+^ mice (Figures 8B,C; KW test: *p* = 0.5626). However, in xCT^−/−^ mice MAG levels were affected by SAS treatment (Figures 8B,D; KW test: *p* = 0.0446). Post-hoc analysis revealed significantly lower levels of MAG immunoreactivity in the spinal cord of xCT^−/−^ mice treated with 320 mg/kg of SAS, but not 160 mg/kg of SAS, compared to saline-treated mice (Dunn’s MTC, saline vs. SAS_160_: *p* = 0.2587, saline vs. SAS_320_: *p* = 0.0339).

**FIGURE 9:**
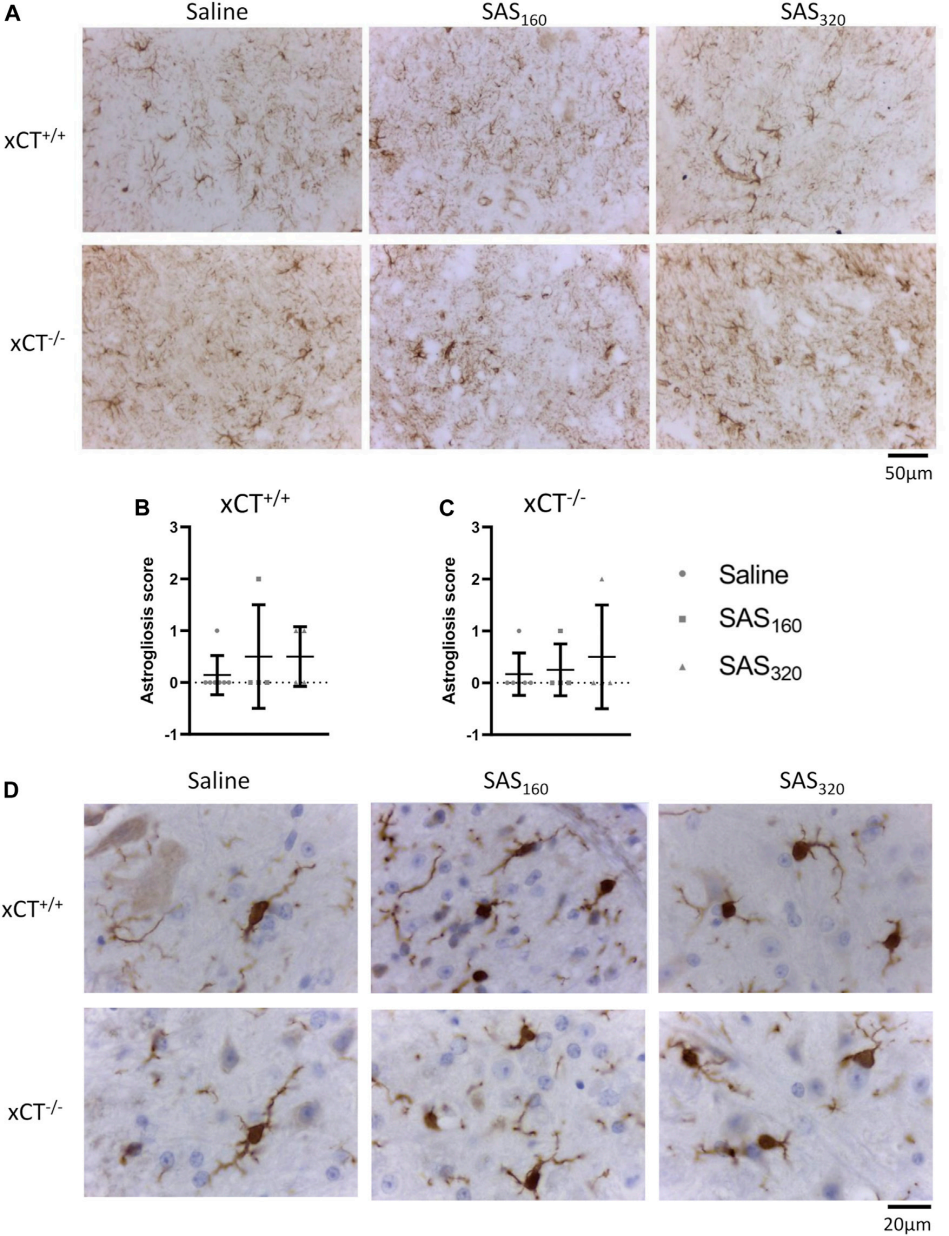
Effects of chronic sulfasalazine (SAS) treatment on astrocytes and microglia in the spinal cord. Representative photomicrographs of GFAP immunohistochemical staining **(A)**. The extent of astrogliosis (normal (0) or presence of slight (1), mild (2) or severe astrogliosis (3)) was quantified in xCT^+/+^ **(B)** and xCT^−/−^ **(C)** mice. Data are presented as mean ± SEM and analyzed using a Fisher's exact test to compare normal GFAP staining (score 0) with signs of astrogliosis (scores 1–3) for each dose of SAS compared to saline. Representative photomicrographs of Iba-1 immunohistochemical staining after a wash-out period of one week **(D)**. One slice of *n* = 4–7 mice/group (for GFAP) or *n* = 5–11 mice/group (for Iba-1) was used.

Finally, with the exception of one or two mice per group, immunohistochemistry for GFAP did not reveal prominent astrogliosis in either xCT^+/+^ (Figures 9A,B; Fisher's exact saline vs. SAS160: *p* > 0.9999, saline vs. SAS_320_: *p* = 0.5227) or xCT^−/−^ mice (Figures 9A,C; Fisher's exact saline vs. SAS160: *p* > 0.9999, saline vs. SAS_320_: *p* > 0.9999), regardless of the treatment. Iba-1 immunohistochemistry showed that microglia of saline-treated as well as those of mice treated with 160 or 320 mg/kg of SAS are in a resting state, independent of genotype (type A microglia, Figure 9D).

## Discussion

Although SAS is not specific in its action, it is one of the most widely used molecules to target system x_c_^−^ in both preclinical (Gout et al., 2001; Ma et al., 2015; Leclercq et al., 2019; Hu et al., 2020) and clinical studies (Shitara et al., 2017; Takeuchi et al., 2014). It has the advantage of being an FDA- and EMA-approved drug for treating Crohn’s disease, ulcerative colitis and rheumatoid arthritis due to its anti-inflammatory effects. While one might expect that this would guarantee the safety of the drug, clinical trials with glioblastoma patients show severe toxicity due to the use of SAS in doses that are needed to achieve inhibition of system x_c_^−^ (Robe et al., 2009). Also our results showed that chronic i. p. administration of SAS -160 mg/kg twice daily and 320 mg/kg once daily- negatively affects the health status of the mice. We observed mortality in both xCT^+/+^ and xCT^−/−^ mice treated with SAS, independent of the dose. Importantly, this effect is independent of its action on system x_c_^−^ as SAS-induced mortality was equally present in mice lacking this transporter. The wide range of adverse effects that are attributed to the use of SAS such as agranulocytosis, haemolytic anaemia, methaemoglobinaemia, hepatotoxicity, nephrotoxicity, neurotoxicity and pulmonary toxicity, might underlie this mortality (Das et al., 1973; Rains et al., 1995; Robe et al., 2009).

We also observed a clear effect of the injections on the body weight of the mice. Chronic i. p. injection with both saline and SAS induced weight loss, which is most probably the result of the chronic stress induced by the daily injections. Repeated vehicle injections were reported to increase the plasma corticosterone levels in BALB/c mice (Drude et al., 2011), which has been shown to affect body weight and food intake (Jeong et al., 2013; Harris, 2015). Also chronic stress itself negatively impacts food intake and body weight in mice (Jeong et al., 2013; Harris, 2015) and rats (Harris et al., 2002; Harris, 2015). Even though all mice lost weight over time, weight loss was more pronounced in the SAS groups, with a prolonged effect in mice treated with the highest dose. This is in accordance to observations in healthy rats that received 200–250 mg/kg of SAS i. p., twice a day for 7 days. While rats normally gain weight over time, this was not the case for the ones treated with SAS (Gout et al., 2001). The SAS-induced decrease in body weight might result from the adverse effects of SAS on the general wellbeing of the mice, presumably regulated via the toxic side effects of sulphapyridine, the main metabolite of absorbed SAS (Sjöquist et al., 1991). Nausea, dyspepsia and abdominal pain as well as loss of appetite and anorexia are typical side effects of SAS in patients with ulcerative colitis, Crohn’s disease and rheumatoid arthritis, and all of these seem to be mediated by sulphapyridine (Das et al., 1973). As sulphapyridine has no inhibitory action on system x_c_^−^ (Gout et al., 2001) and given the same decrease in body weight in xCT^−/-^ mice, we are confident that none of these effects are mediated via chronic inhibition of this transporter.

On top of the mortality and the effect on body weight, this study shows a clear effect of SAS on body temperature shortly after injection. While in normal conditions a stressful event -such as an injection- would induce hyperthermia (Olivier et al., 2003), mice treated with SAS showed a strong but temporary hypothermic reaction that is independent of the presence of xCT. This could be due to the chronic inescapable stress experienced by the daily injections (Oka, 2018). However, saline-treated mice showed a normal stress-induced hyperthermic reaction even though they experienced the same level of stress, suggesting a specific effect of SAS either on the stress reaction of the mice or on their thermoregulation. Despite the general hypothesis that stress-induced hyperthermia is cytokine- and PGE_2_-independent, some studies do show an involvement of PGE_2_ in this phenomenon (Morimoto et al., 1991; Parrott and Lloyd, 1995; Oka, 2018). Since SAS, and more specifically the metabolite mesalazine (Sjöquist et al., 1991), is able to decrease the PGE_2_ production (Karagozian & Burakoff, 2007), this might abolish the PGE_2_-induced hyperthermia. As this is entirely hypothetical and cannot fully explain the hypothermic reaction after chronic SAS treatment, this phenomenon requires further investigation.

While SAS-induced chronic inhibition of system x_c_^−^ was reported to induce myelin degeneration in the white matter of the spinal cord (Soria et al., 2016), we could not observe spinal cord damage after chronic SAS administration to xCT^+/+^ or xCT^−/−^ mice. Four weeks of SAS treatment did not influence the number of neurons or the myelin protein content in the spinal cord. However, whereas there was no decrease in MBP or MAG immunoreactivity in our study, Soria and colleagues detected a strong reduction in MBP and abnormalities in myelin folding in spinal cord and sciatic nerve samples using a similar dose of SAS (320 mg/kg) and the same treatment period (Soria et al., 2016). This discrepancy could possibly be related to the strain of mice that has been used in the latter study. It should be noted that we did observe decreased MAG reactivity in xCT^−/−^ mice treated with SAS. Obviously, this effect cannot be mediated via chronic inhibition of system x_c_^−^, as these mice lack functional system x_c_^−^. Moreover, the absence of activated microglia and the lack of astrogliosis in the spinal tissue of all groups studied, further indicate that in our hands, chronic SAS treatment does not induce any cell damage in the spinal cord. This is in line with the *in vitro* observation that aminoadipic acid-induced inhibition of system x_c_^−^ does not influence the viability of oligodendrocytes (Domercq et al., 2007) and further supported by an *in vivo* report on reduced demyelination in the EAE model for MS after SAS treatment (Evonuk et al., 2015).

We showed that both saline- and SAS-treated mice tend to cover a decreased distance in the OF arena over the time of testing, which is most probably due to a habituation process (Sousa et al., 2006). The first time the mice encounter the OF arena, SAS treatment decreased the distance that both xCT^+/+^ and xCT^−/−^ mice travelled, again indicating that this effect is not mediated by inhibition of system x_c_^−^. This is further supported by the fact that naive xCT^−/−^ mice do not show abnormalities in spontaneous behavior and walk the same distance in the OF test compared to naive xCT^+/+^ mice (Bentea et al., 2015). As described above, SAS did not cause abnormalities in the spinal cord, making it unlikely that the effect on the distance walked in the OF results from toxicity on spinal motor pathways. Moreover, this effect disappeared after a wash-out period of one week. Therefore, the fact that mice treated with SAS travelled significantly less compared to saline injected mice the first time they perform the OF test, is most probably not reflecting a motor problem, but rather linked to motivation to explore the maze.

I.p. injection of a low dose of SAS (8 or 16 mg/kg) induced an anxiogenic effect in rats as evaluated using the OF and the EPM (Lutgen et al., 2014). However, in our study, SAS treatment did not have an effect on anxiety like-behavior in xCT^+/+^ mice in the same behavioral setups. We rather anticipated anxiolytic effects after chronic inhibition of system x_c_^−^ using SAS, as seen in naive mice lacking functional system x_c_^−^ (Bentea et al., 2015). Yet, in the current study also the saline-injected xCT^−/−^ mice did not show a convincing anxiolytic phenotype when compared to saline-injected xCT^+/+^ mice, and even a borderline-significant anxiogenic phenotype in the EPM (Mann-Whitney test: cumulative duration in the center of the OF: w2: *p* = 0.6047, w3: *p* = 0.04679, w4: *p* = 0.2895, w5: *p* = 0.9725; time spent in the open arms of the EPM: *p* = 0.05116). It is possible that the time of testing was too short to pick up differences in anxiety-like behavior as the anxiolytic effect in naive xCT^−/−^ mice was only present when the mice were allowed to explore the OF arena for a longer time (60 min) (Bentea et al., 2015). Moreover, differences in this type of behavior might be masked by the chronic stress of the daily injections. Studies showed that saline-treated rodents already exhibit a stressed and anxious profile (Lapin, 1995) and that the time spent in the center of the OF can be influenced by the handling method (Gouveia and Hurst, 2019). Mice picked up by their tail, the method used to inject the mice in our study, spent less time in the center of the OF compared to mice being picked up in a tunnel (Gouveia and Hurst, 2019). Unexpectedly, we showed that treatment of xCT^−/−^ mice with 320 mg/kg of SAS induced a decreased time spent in the center of the OF, which would indicate an anxiogenic effect of SAS in these mice that is independent of system x_c_^−^.

Inhibition of system x_c_^−^ not only has anxiolytic, but also antidepressant potential, as evidenced by naive as well as LPS-injected xCT^−/-^ mice showing a decreased immobility-time in the MTS and in the forced swim test (Bentea et al., 2015; Albertini et al., 2018). Moreover, tumor-inoculated BALB/c mice showed reduced tumor-associated depressive-like behavior after chronic inhibition of system x_c_^−^ using SAS (Nashed et al., 2017). However, in the current work, saline-injected xCT^−/−^ mice did not display an anti-depressive profile compared to xCT^+/+^ mice (Mann-Whitney test: immobility time in the MTS: *p* = 0.1230), and we could not induce this phenotype by chronic inhibition of system x_c_^−^ using SAS in xCT^+/+^ mice. In line with the data on anxiety-like behavior, the results on depressive-like behavior can be influenced by the chronic stress (Dunn and Swiergiel, 2008), in accordance with our previous observation that xCT^−/−^ mice lose their anti-depressive like phenotype when they are subjected to stress induced by chronic corticosterone administration (Demuyser et al., 2019).

## Conclusion

This work shows adverse effects of chronic SAS treatment on the wellbeing of the mice, in the absence of spinal cord damage, compromised motor function, anxiety- or depressive-like behavior. All effects of chronic SAS administration detected in this study are, however, independent of its function as an inhibitor of system x_c_^−^ and thus originate from (toxic) off-target effects of the molecule or its metabolites. While a lot of progress has been made in the search for new molecules that selectively act on system x_c_^−^ (Patel et al., 2019; Nehser et al., 2020), this study emphasizes the need for further research in this area to allow safe targeting of system x_c_^−^ in diverse neurological disorders and cancer types.

## Data Availability

The raw data supporting the conclusions of this article will be made available by the authors, without undue reservation.

## Abbreviations

EPM, elevated plus maze; i.p, intraperitoneal; KW, Kruskal-Wallis; LPS, lipopolysaccharide; MAG, myelin-associated glycoprotein; MBP, myelin basic protein; MCT, multiple comparisons test; MS, multiple sclerosis; MSRT, matched-pairs signed rank test; MTS, mouse tail suspension; OF, open field; SAS, sulfasalazine; SEM, standard error of the mean; TBS, Tris-buffered saline.
